# Clonal abundance patterns in hematopoiesis: Mathematical modeling and parameter estimation

**DOI:** 10.3389/fsysb.2023.893366

**Published:** 2023-02-09

**Authors:** Yunbei Pan, Maria R. D’Orsogna, Min Tang, Thomas Stiehl, Tom Chou

**Affiliations:** ^1^ Department of Computational Medicine, UCLA, Los Angeles, CA, United States; ^2^ Department of Mathematics, California State University at Northridge, Los Angeles, CA, United States; ^3^ Institute of Natural Sciences, Shanghai Jiaotong University, Shanghai, China; ^4^ Institute of Computational Biomedicine, RWTH Aachen University, Aachen, Germany; ^5^ Department of Mathematics, UCLA, Los Angeles, CA, United States

**Keywords:** stem cells, hematopoiesis, barcodes, clonal tracking, differentiation

## Abstract

Hematopoiesis has been studied *via* stem cell labeling using barcodes, viral integration sites (VISs), or *in situ* methods. Subsequent proliferation and differentiation preserve the tag identity, thus defining a clone of mature cells across multiple cell type or lineages. By tracking the population of clones, measured within samples taken at discrete time points, we infer physiological parameters associated with a hybrid stochastic-deterministic mathematical model of hematopoiesis. We analyze clone population data from Koelle et al. (Koelle et al., 2017) and compare the states of clones (mean and variance of their abundances) and the state-space density of clones with the corresponding quantities predicted from our model. Comparing our model to the tagged granulocyte populations, we find parameters (stem cell carrying capacity, stem cell differentiation rates, and the proliferative potential of progenitor cells, and sample sizes) that provide reasonable fits in three out of four animals. Even though some observed features cannot be quantitatively reproduced by our model, our analyses provides insight into how model parameters influence the underlying mechanisms in hematopoiesis. We discuss additional mechanisms not incorporated in our model.

## Introduction

Hematopoiesis, the process by which hematopoietic stem cells (HSCs) generate all mature blood cells in an animal through proliferation and differentiation plays a crucial role in an organism’s immune response and maintaining overall homeostasis. Estimates of the number of actively cycling HSC range from 50000–200000 in humans (Lee-Six et al., 2018) and approximately 5,000 in mice (Busch et al., 2015; Mayle et al., 2015). It is well-known that these small numbers of hematopoietic stem cells can generate 10^10^–10^12^ cells of multiple cell types daily, over an organism’s lifetime (Fliedner, 2002; Doulatov et al., 2012). Understanding the mechanisms of hematopoiesis can help guide clinical treatment, especially those related to bone marrow transplantation and in the context of blood cancers (Mendelson and Frenette, 2014; Busch et al., 2015; Goyal et al., 2015).

HSCs are often quiescent (Seita and Weissman, 2010), making them hard to track *in vivo* and difficult to control *in vitro*. Thus, the HSC dynamics *in vivo* can only be straightforwardly interrogated through analysis of populations of more downstream progenitors and differentiated blood cells (Bystrykh et al., 2012). One way to quantitatively probe the hematopoiesis process is the labeling of multipotent HSCs by tagging their genomes. The tags can take the form of viral integration sites or barcodes (Grosselin et al., 2013; Kim et al., 2014; Wu et al., 2014; Biasco et al., 2016; Koelle et al., 2017). In a typical *in vivo* experiment, CD34^+^ stem cells are extracted, tagged, and then autologously transplanted back into the animal, typically a mouse or a rhesus macaque. CD34^+^ cells contain HSCs as well as early hematopoietic stem and progenitor cells (HSPCs), with a wide range of estimated relative proportions (Corso et al., 2005; Attar, 2014; Parmentier et al., 2020). The downstream progenitor and mature cells that derive from proliferation of HSCs of a particular tag will form a clone of cells that share the same tag. Clonal tracking of cell tags is thus a powerful tool for interrogating the differentiation process during hematopoiesis (Lyne et al., 2018; Challen and Goodell, 2020; Cordes et al., 2021). For example, the abundances of the different tags that appear in the different types of mature cells can shed light on the branching structure of differentiation and on proliferation dynamics, particularly when coupled with mathematical models and/or simulations (Stiehl and Marciniak-Czochra, 2011; Sun and Komarova, 2012; Székely et al., 2014; Höfer and Rodewald, 2016; Xu J. et al., 2018).

Clonal tracking in mice (Copley et al., 2012; Sun et al., 2014) has revealed the timescales of repopulation dynamics under homeostasis and after bone marrow transplantation (Muller-Sieburg et al., 2012; Verovskaya et al., 2013; Busch et al., 2015), but typically involves very few clones that cover only a small fraction of the HSC population. To transplant many HSC clones in order to see patterns of how clones are distributed during hematopoiesis requires experiments on animals larger than mice.

Transplant experiments in rhesus macaque on the other hand allow for hundreds or thousands of clones to be engrafted into an organism that exhibits population levels and timescales closer to those in humans. One experiment in rhesus macaque involved tracking HSC clonal dynamics of lentivirus-tagged HSCs and early progenitor cells (HSPCs), and following hematopoiesis over a time period comparable to the animal’s life-span (Kim et al., 2010, 2014). Here, CD34^+^ HSPCs from the bone marrow, which include various progenitor cells, were marked *via* the integration of a lentivirus vector with an accompanying green fluorescent protein (GFP) tag at random viral integration sites (VISs). After sublethal myeloablative irradiation to eliminate a substantial number of cells in the bone marrow, the tagged HSPCs were autologously transplanted. If these cells divide and differentiate after transplantation, their progeny will inherit the unique VISs. Sampling and sequencing of these mature cells indicates which ones are descendants of a founder HSC. Data collected from four macaques over 14 years were analyzed showing how bias towards the lymphoid or myeloid differentiation branches changes over time. More detailed analyses were also performed in order to connect clonal patterns during hematopoiesis with a mathematical model that describes how self-renewal, differentiation, and subsampling of a multiclone population affects clone abundances and their fluctuations across time (Goyal et al., 2015; Xu S. et al., 2018). By fitting a simple mechanistic model to abundances of hundreds to thousands of clones, random initial differentiation events that each led to a subsequent burst of mature cells was proposed as a mechanism to explain observed population fluctuations. The number of generations *L* that progenitor cells traverse along a differentiation pathway (lineage) before terminal differentiation was also estimated to be *L* ∼ 24 for the granulocyte lineage (Xu S. et al., 2018). To obtain this result, a mean-field model for HSC self-renewal was developed and applied to experimental data on granulocytes, using only the mean and variance of clone populations in the data fitting.

In this paper, we improve on the model used in (Xu S. et al., 2018) by developing a framework that can explain population transients and that can predict the density of the number of clones with respect to mean clone sizes. Instead of analyzing VIS data from (Kim et al., 2010, 2014), we consider the barcode data from (Wu et al., 2014; Koelle et al., 2017). In the latter experimental studies, replication-incompetent HIV-derived lentiviral barcoding vectors were used to tag HSCs that were transplanted into four rhesus macaques. The barcode consists of a six base-pair library identification and a 35 base pair high-diversity cellular barcode. As with the VIS experiments, barcoded cells were reinfused in the animals after myeloablative total-body irradiation. Purified samples of blood cells were then subject to low-cycle PCR amplification with the two primers bracketing the barcode. This barcoding approach provides more precise quantification relative to other clonal tracking protocols such as VIS (Kim et al., 2014) and transposon tagging (Sun et al., 2014) approaches. Thus, we will analyze the barcoding data *via* a mathematical model with the goal of more accurately estimating physiological parameters such as HSC carrying capacity, progenitor cell division rates, and progenitor cell proliferative potential for the granulocyte cell lineage. Although clonal structure of mature cells of different lineages, such as T, B, monocytes, and NK cells, were quantified in (Wu et al., 2014; Koelle et al., 2017), lymphocyte maturation is more complex, involving additional intermediate steps and subsequent immune signaling and mature cell proliferation. Thus, we focus on the simpler and abundant mature granulocyte population (Bystrykh et al., 2012).

In the following Materials and Methods section, we briefly describe the raw data and present the mathematical model. In the Data Analysis and Results section, we describe how measured clone data is compared to predicted clone abundances and show that minimization of the difference leads to reasonable estimates of parameter estimates. Finally, in the Discussion and Conclusions, we provide qualitative insight into how model parameters affect the predicted clonal patterns and discuss further improvements and potential new modeling directions.

## Materials and methods

In this section, we describe information extracted from the granulocyte abundance data in (Koelle et al., 2017) and the mathematical model used to describe this data. The experimental parameters associated with the experiments are listed in Table 1, which lists the number of cells (tagged and untagged) transplanted, the barcode library size used, and the total number of different barcodes observed across all samples of all lineages for each animal. These values inform us on the typical magnitude of experimental parameters to which our subsequent model must conform. In Table 2, we list parameters used in our mathematical model as determined either from experimental data or through estimates.

**TABLE 1:** Transplant parameters. The initial transplant populations for the four animals ZH33, ZG66, ZH19, and ZJ31. The total library size for the cell preparation was in the range *C*_*L*_ = 53319 − 109085. The total number of cells injected was *H* = 2.3 × 10^7^ − 4.8 × 10^7^, of which H* = 8.0 × 10^6^ − 1.67 × 10^7^ were barcoded (corresponding to 23%–35% GFP+ labeling). Across all peripheral blood samples and cell lineages, the total number of barcodes detected in each animal was in the range ${\hat{C}}_{s}=21450-62354$ , *i.e.*, roughly half of injected HSC barcodes were detected in the peripheral blood samples. Among granulocytes, the total sampled richness (across all time points) ranged from 2660 − 32363.

Variable Animal	Library size *C*_*L*_	Injected cells *H*	Injected GFP+ H*	Total *C*_s_	*C*_s_ (grans)
ZH33	63469	3.2 × 10^7^	1.11 × 10^7^	25325	9221
ZG66	53613	4.8 × 10^7^	1.67 × 10^7^	21450	2660
ZH19	53319	4.8 × 10^7^	1.1 × 10^7^	31929	10964
ZJ31	109085	2.3 × 10^7^	8.0 × 10^6^	62354	32363

**TABLE 2:** Overview of variables and parameters. Parameters and variables and their estimated values if known. Some values need to be calculated from our model and are denoted “calc.,” while others need to be self-consistently estimated. For example, from GFP tagging, the fraction of tagged HSCs is approximately $\sum_{i=1}^{C_{h}}h_{i}\left(0\right)/\sum_{i=0}^{C_{h}}h_{i}\left(0\right)\approx 15-35\%$ but can slowly vary in time. Values relating to sampled cell populations are derived from animal ZH33 in the experiment of (Koelle et al., 2017). HSC proliferation and death rates have been estimated in (Shepherd et al., 2007) and (Catlin et al., 2011). Numbers specific to granulocytes are indicated as such.

Variables/Parameters	Definition	Value
*t*_*j*_, *j* = 1, …, *J*	Sampling time points	∼month
${\hat{s}}_{i}\left(t_{j}\right),\;i=1,\ldots ,C_{s}$	No. of cells with tag *i* in sample drawn at *t*_*j*_ (data)	$∼0-10^{4}$
${\hat{s}}_{i}=\frac{1}{J}\sum_{i=1}^{C_{s}}{\hat{s}}_{i}\left(t_{j}\right)$	Mean no. of cells with tag *i* in sample (data)	$∼0-10^{4}$
$\hat{S}\left(t_{j}\right)=\sum_{i=1}{\hat{s}}_{i}\left(t_{j}\right)$	Total no. of tagged granulocytes in each sample at *t*_*j*_ (data)	$∼2\times 10^{6}$
${\hat{C}}_{s}\left(t_{j}>2\;\text{months}\right)$	Total no. of clones (richness) in sample *j*	∼1000 (grans)
${\hat{C}}_{s}^{>2}$	Total richness across all *t* > *t*_2_	2,335 (grans)
*h*_0_	Untagged HSCs in bone marrow (model)	unknown, $∼10^{4}$
*h*_*i*_, *i* = 1, …, *C*_h_	HSCs with barcode *i* in BM (model)	1–1,000
$n_{i}^{\left(\ell \right)}\left(t\right)$	No. of *ℓ* ^th^-generation progenitor cells with tag/barcode *i* (model)	calc
*m*_*i*_(*t*)	No. mature cells with tag *i* (model)	calc
*s*_*i*_(*t*)	No. of cells with tag *i* in sample (model)	calc
*K*	HSC niche carrying capacity (model)	inferred, 10^4^–10^5^
*C*_h_(*t*)	Total no. of engrafted clones (model)	unknown, $∼10^{4}$
*C*_s_(*t*)	Total no. of clones sampled at *t* (model)	simulated
*r*_h_(0)	Intrinsic HSC self-renewal rate	≫ 0.01/day
*μ*_h_	HSC death rate	<0.01 /day
*α*	HSC differentiation rate	10^–3^ − 0.02/day, inferred
*r*_n_	Progenitor cell division rate	1–5/day
*μ*_n_	Progenitor cell death rate	unknown, ∼0 /day
*ω*	Progenitor cell terminal differentiation rate	unknown, $<r_{n}$
*L*	Proliferative potential of progenitor cells	inferred, L* = 22 (grans)
*μ*_m_	Mature cell death rate	0.185/day (grans)
*η*	Average sample fraction	$∼10^{-5}-10^{-4}$
*η*(*t*_*j*_)	Fraction of sample *j*	$∼10^{-5}-10^{-4}$

### Measured quantities

**FIGURE 1:**
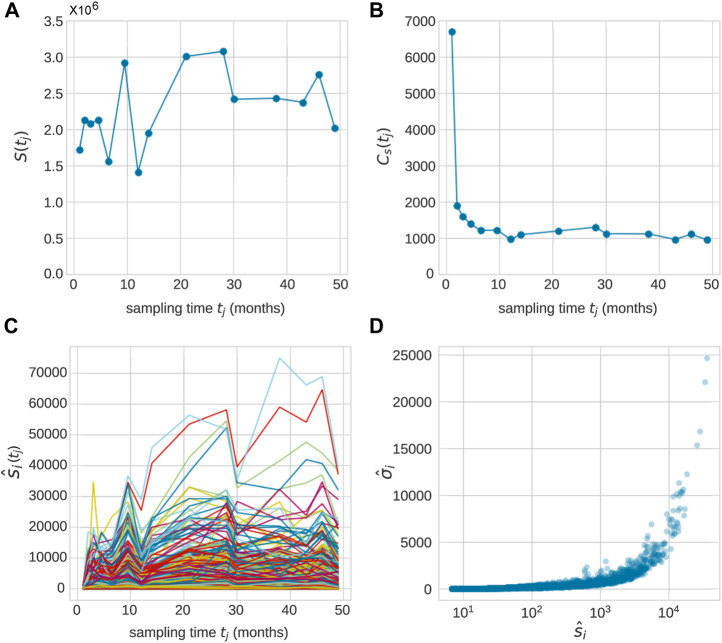
After transplantation, peripheral blood samples were taken across *J* time points *t*_*j*_, *j* = 1, …, **(J)**. Typically, measurements were taken over 20–49 months and *J* = 10 − 15. **(A)** The total population $\hat{S}\left(t_{j}\right)$ of granulocytes sampled from animal ZH33 (Koelle et al., 2017) at times *t*_*j*_ = (1, 2, 3, 4.5, 6.5, 9.5, 12, 14, 21, 28, 30, 38, 43, 46, 49) months. **(B)** The total richness in each sample, ${\hat{C}}_{s}\left(t_{j}\right)$ . The richness at the first two time samples are large (as we shall see, due to transplantation of barcoded progenitor cells). After the first two time points, where the richness will arise from the richness of the transplanted HSCs, the typical richness at each time point ${\hat{C}}_{s}\left(t_{j}≳2\right)\approx 1000$ , while the richness across all *J* − 2 time points (for *t*_*j*_ > 2 months) is ${\hat{C}}_{s}^{>2}=2335$ . Across *all* *J* *time points*, 9,221 unique granulocyte clones were detected (out of a total of 25325 across *all cell types*). The individual clone abundances in the sampled granulocyte population are shown in **(C)** where the abundances of clone *i* in a sample taken at time *t*_*j*_ are denoted by ${\hat{s}}_{i}\left(t_{j}\right)$ . The mean and standard deviation ${\hat{\sigma }}_{i}$ of the abundances of all clones across all sampling times are calculated using Eq. 1 and scatter-plotted in **(D)**. Each point represents one of the 2,335 detected granulocyte clones.

First, we consider the observed data associated with each animal (Koelle et al., 2017), as shown in Figure 1. Granulocytes in blood samples drawn from each animal at times points *t*_*j*_, *j* = 1, 2, …, *J* are sequenced and clonal (barcode) abundances tabulated. The total abundance (number of mature cells of a given cell type), $\hat{S}\left(t_{j}\right)$ , and the richness ${\hat{C}}_{s}\left(t_{j}\right)$ (the total number of different barcodes detected in each sample) are also recorded and plotted in Figures 1A, B. In this study, $\hat{S}\left(t_{j}\right)$ denotes the total measured number of granulocytes (barcoded and unbarcoded) in a sample taken at time *t*_*j*_. The fluctuations of $\hat{S}\left(t_{j}\right)$ and ${\hat{C}}_{s}\left(t_{j}\right)$ across *t*_*j*_ may arise from varying sampled sizes across time points and/or fluctuations in the state of the animal.

An example of the abundances of each clone within the granulocyte population from Koelle et al. (Koelle et al., 2017) is shown in Figure 1C. In these experiments, tagged stem cells are transplanted back into a rhesus macaque at *t* = 0 so that initially each clone consists of a single cell. A series of 1 ≤ *j* ≤ *J* samples are taken at time *t*_*j*_ after implementation, yielding a set of mature cells. We denote the abundance of clone *i* (among granulocyte cells) in the sample taken at time *t*_*j*_ after transplantation as ${\hat{s}}_{i}\left(t_{j}\right)$ . The *J* measurements allow each clone of a particular mature cell type *i* to be characterized by a mean ${\hat{s}}_{i}$ and variance ${\hat{\sigma }}_{i}^{2}$ defined by

$$\begin{array}{cc} {\hat{s}}_{i}= & \frac{1}{J}\overset{J}{\underset{j=1}{\sum }}{\hat{s}}_{i}\left(t_{j}\right)\\ {\hat{\sigma }}_{i}^{2}= & \frac{1}{J}\overset{J}{\underset{j=1}{\sum }}{\left({\hat{s}}_{i}\left(t_{j}\right)-{\hat{s}}_{i}\right)}^{2}. \end{array}$$ (1)

Note that the total measured population of any cell type $\hat{S}\left(t_{j}\right)=\sum_{i=1}^{C_{s}}{\hat{s}}_{i}\left(t_{j}\right)$ . A scatter plot of ${\hat{\sigma }}_{i}$ versus ${\hat{s}}_{i}$ for all clones detected in a sample of granulocytes is shown in Figure 1D.

For each clone at $\left({\hat{s}}_{i},{\hat{\sigma }}_{i}\right)$ we can evaluate the local density $\hat{\rho }$ , the number of clones within some size window. This density can be viewed as the concentration of data points shown in Figure 1D as a function of mean clone size $\hat{s}$ , and will be constructed using kernel density estimation (Rosenblatt, 1956; Parzen, 1962) of the data points in $\left(\hat{s},\;\hat{\sigma }\right)$ space. The unknown density function $\hat{\rho }$ is obtained by concatenating isotropic Gaussian kernel functions about each point and using an optimal, common bandwidth parameter, typically chosen as the value that minimizes the mean integrated squared error, or Kernel Density Estimation (KDE) (Silverman, 1986). The reconstructed density function can be thought of as a probability that a random clone arises in the volume (*s*, *s* + d*s*) × (*σ*, *σ* + d*σ*). For each clone at $\left({\hat{s}}_{i},{\hat{\sigma }}_{i}\right)$ we can evaluate the local density $\hat{\rho }$ .

In the remainder of this paper we will develop a mathematical model that we can simulate to generate total populations, clonal populations, and their associated attributes (*s*_*i*_, *σ*_*i*_, *ρ*_*i*_). Note that while there are many existing mathematical models of hematopoiesis (Colijn and Mackey, 2005; Peixoto et al., 2011; De Souza and Humphries, 2019), they describe only time variations in total populations, rather than that of lower-population, individual clones. We will tune parameters of the model so that its predictions provide a reasonable match to the aforementioned measurements, paying particular attention to clone abundances and clone size variability.

### Mathematical modeling

Our mathematical model incorporates known and accepted features of hematopoiesis. Three main cell compartments are considered: hematopoietic stem cells (HSCs), transit amplifying progenitor cells, and peripheral mature cells. Although the stem cell population in bone marrow is large and can be described using a deterministic model, the initial populations within each clone are small and require a discrete stochastic description. We will then assume that a small sample of mature cells is drawn from the animals at times *t*_*j*_, *j* = 1, 2, …, *J* and sequenced.

We first describe the initial conditions including the number of HSCs and HSPCs injected into each animal. As listed in Table 1, *H* is the total number of HSCs injected into each animal, among which *H*_0_ are untagged and *H*_*i*_ ≪ *H*_0_ contain barcode 1 ≤ *i* ≤ *C*_*H*_ (and are GFP+). The total tagged HSC population is $H^{*}\equiv \sum_{i=1}^{C_{H}}H_{i}$ so that *H* = *H*_0_ + H*. The richness *C*_*H*_ ≲ *C*_*L*_ is the number of barcodes transferred into the animal, which is comparable to the richness of the barcode library *C*_*L*_ used in each experiment. Since *H*_0_ ∼ 10^7^ ≫ *H*_*i*_, we will consider the probability distribution of only the tagged populations, which is described by the multinomial

$$P\left(H\right)=\left(H^{*}\right)!\overset{C_{H}}{\underset{i=1}{\prod }}{\left(\frac{1}{C_{H}}\right)}^{H_{i}}\frac{1}{H_{i}!},$$ (2)

where $H\equiv \left(H_{1},H_{2},\ldots ,H_{C_{H}}\right)$ and H* is the total number of GFP+ (barcoded) cells. Specifically, for animal ZH33 studied in (Koelle et al., 2017)) *H* ≈ 3 × 10^7^, H*/*H* ≈ 0.35, $\sum_{i=1}^{C_{H}}H_{i}\approx 1.1\times 10^{7}$ , *C*_*H*_ ≲ *C*_*L*_ ≈ 6 × 10^5^. Thus, the typical *H*_*i*_ ≈ H*/*C*_*H*_ ∼ 180.

A certain fraction *η*_0_ of the *H* HSCs home into the bone marrow, successfully engraft, and subsequently actively self-renewal and/or differentiate. Engrafted HSC populations are defined by $h\left(0\right)=\left(h_{1}\left(0\right),h_{2}\left(0\right),\ldots ,h_{C_{h}\left(0\right)}\;\left(0\right)\right)$ , where the richness of engrafted HSCs in the bone marrow is *C*_h_ ≲ *C*_*H*_. Transplantation efficiencies are typically single-digit percentages (Abbuehl et al., 2017; Radtke et al., 2020) and transplanted CD34^+^ cells contain significant numbers of progenitor cells. Thus, the fraction *η*_0_ ≪ 1; if *η*_0_ is sufficiently small (approximately ≲ 1/180), then we can safely assume that the initial clone populations in bone marrow are represented by very few cells. For simplicity, we approximate *h*_*i*_(0) ≈ 1. Even if *η*_0_≰1/180, most barcodes will be represented by very few cells. We have verified that an initial condition in our model that allows for, say, some *h*_*i*_(0) = 2, 3 does not qualitatively affect the mature cell populations.

The random selection of cells into the bone marrow can be thought of as a sampling (without replacement) process. Including the untagged population, the probability distribution of engrafted cells resulting from the injected tagged cell population $H=\left(H_{1},H_{2},\ldots H_{C_{H}}\right)$ is given by

Ph0|H=1H*h*0∏i=1CHHihi0, (3)

where H* and $h^{*}\left(0\right)=\sum_{i=1}^{C_{H}}h_{i}\left(0\right)$ are the total initial numbers of barcoded injected cells and engrafted barcoded HSCs, summed over all clones. Note that the number of untagged transplanted cells *h*_0_(0) ≫ 1 is large so that we can approximate it by its deterministic value *h*_0_(0) ≈ *η*_0_ *H*_0_.

To extract the overall probability of initial condition **h**(0), we average Eq. 3 over the prior $P\left(H_{1},\ldots ,H_{C_{H}}\right)$ and find

$$P\left(h\left(0\right)\right)=\underset{H}{\sum }P\left(h\left(0\right)|H\right)P\left(H\right)=h^{*}\left(0\right)!\overset{C_{H}}{\underset{i=1}{\prod }}{\left(\frac{1}{C_{H}}\right)}^{h_{i}\left(0\right)}\;\;\;\;\frac{1}{h_{i}\left(0\right)!},\;h^{*}\left(0\right)\equiv \overset{C_{H}}{\underset{i=1}{\sum }}h_{i}\left(0\right).$$ (4)

Besides the initial condition *h*_*i*_(*t* = 0) = 1, the initial number of untagged HSCs *h*_0_(*t* = 0) is related to the transplantation efficiency and is generally unknown. Barcodes are associated with a GFP tag and the initial fraction of sampled cells that are GFP+ is ∼35% . Since we assume a neutral model, it is reasonable to assume that the fraction of injected tagged cells H*/*H* is equivalent to the fraction of tagged cells in the engrafted population $\sum_{i=1}^{C_{h}\left(0\right)}h_{i}\left(0\right)/\sum_{i=0}^{C_{h}\left(0\right)}h_{i}\left(0\right)\approx 0.35$ (although this ratio slowly decreases *via* extinction). The precise richness of HSC population in stem cell niche, *C*_h_(*t*) < *C*_*H*_ is also unknown, but except for fluctuations, has a lower bound of ${\hat{C}}_{s}$ , the total number of unique clones detected across all samples across all cell types. Thus, we take $h\left(0\right)=\sum_{i=0}^{C_{h}\left(0\right)}h_{i}\left(0\right)=h_{0}\left(0\right)+C_{h}\left(0\right)\approx C_{h}\left(0\right)/0.35$ .

Self-renewal, death, and differentiation into progenitor cells all contribute to the stochastic dynamics of *h*_*i*_. Although the *total* HSC population in the niche $h\left(t\right)\approx \sum_{i=0}^{C_{h}\left(t\right)}h_{i}\left(t\right)$ is large and can be approximated deterministically, the HSC population of each clone *h*_*i*_(*t*) may be small and must be treated stochastically. Under our neutral assumption, the intrinsic self-renewal rate *r*_h_ of HSCs does not depend on the barcode identity *i*. Since HSCs reside in niches in the bone marrow that place limits on growth, we assume the HSC proliferation rate follows a linearly decreasing form defined by a carrying capacity and the engrafted HSC population *h*(*t*)

$$r_{h}\left(h\left(t\right)\right)=r_{h}\left(0\right)\left(1-\frac{h\left(t\right)}{K}\right)\;\;\;\;\text{, }\;\;\;h\left(t\right)\equiv \overset{C_{h}}{\underset{i=0}{\sum }}h_{i}\left(t\right),$$ (5)

where *r*_h_(0) is the intrinsic proliferation rate of a single, isolated HSC. Note that the untagged HSCs are included through *h*_0_. Finally, we assume that HSCs die at rate *μ*_h_ and differentiate at rate *α* and that these rates, like the growth rate in Eq. 5, do not depend on barcode identity.

As shown in the Supplementary Material, the richness *C*_h_(*t*) may progressively decrease from random HSC death and extinction and can be estimated by solving for the stochastic birth-death process (neglecting outflux from differentiation) and using generating functions to find

$$E\left[C_{h}\left(t\right)\right]\approx \frac{C_{h}\left(0\right)}{\psi \left(t\right)+\phi \left(t\right)},$$ (6)

where

$$\psi \left(t\right)\equiv e^{-\int_{0}^{t}\left[r_{h}\left(t^{\prime }\right)-\mu_{h}\right]dt^{\prime }},\;\phi \left(t\right)\equiv \int_{0}^{t}r_{h}\left(t^{\prime }\right)\psi \left(t^{\prime }\right)dt^{\prime }.$$ (7)

In this expression, *r*_h_(*t*) is approximated by $r_{h}\left({\bar{h}}_{0}\left(t\right)+{\bar{h}}^{*}\left(t\right)\right)$ , where ${\bar{h}}_{0}+{\bar{h}}^{*}\left(t\right)$ is given by the explicit solution to the deterministic birth-death process with carrying capacity *K*. Thus, given *C*_h_(0), *r*_h_(0), *K*, and *μ*_h_ determine how the expected richness decreases. Henceforth we treat *C*_h_(*t*) in our model as the expected value $E\left[C_{h}\left(t\right)\right]$ derived from our stochastic birth-death model, *i.e.*, we use *C*_h_(0)/(*ψ*(*t*) + *ϕ*(*t*)) as the model for *C*_h_(*t*).

**FIGURE 2:**
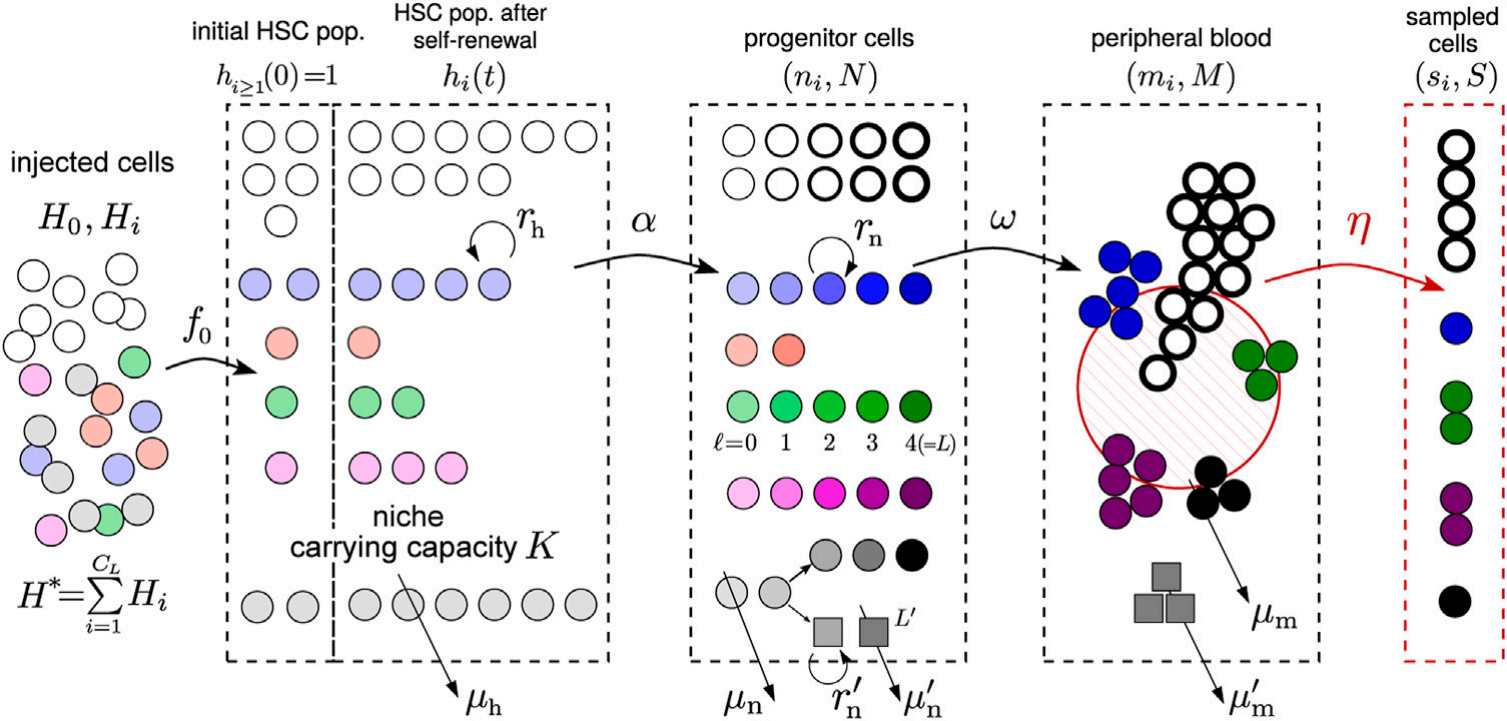
Schematic of the hybrid stochastic-deterministic model. Tagged (barcoded) stem cells are transplanted into an animal initially with one cell (*h*_*i*_(*t* = 0) ≈ 1) per clone. These cells, together with the untagged ones (*h*_0_(*t* = 0) ≫ 1) then undergo self-renewal and death, at rates $r_{h}\left(h\left(t\right)=\sum_{i=0}h_{i}\left(t\right)\right)$ and *μ*_h_, respectively, in the bone marrow. HSCs in all clones are also assumed to undergo asymmetric differentiation with rate *α*, forming a zeroth-generation progenitor cells. The population of *ℓ* ^th^-generation (or stage) progenitor cells, denoted $n_{i}^{\left(\ell \right)}$ , further symmetrically differentiate with each division, up to a maximum of *ℓ* = *L* generations. The final-generation cell in clone *i* with population $n_{i}^{\left(L\right)}$ can then undergo terminal differentiation at rate *ω* to form mature, circulating peripheral blood cells. Mature cells at population *m*_*i*_ are then randomly sampled (with sampling fraction *η* and generating a sample population *s*_*i*_) and sequenced. We wish to infer some of the parameters of the model by comparing the predicted means, standard deviations, and clone number densities with those from data (Figure 1). Lineage differentiation is schematically shown as a splitting of the grey clone between generations *ℓ* = 1 and *ℓ* = 2, where a new cell type (squares) branches off. The division and death rates of progenitor cells in this new lineage, $r_{n}^{\prime }$ and $\mu_{n}^{\prime }$ , may be different, as may the maximum number of generations *L*′. The mature cells turn over with rate a *μ*_m_ that may depend on lineage (but not clone identity within each lineage). In this paper, we assume that the lineages diverge at the zeroth-generation progenitor cell and analyze the model after the first differentiation step (rate *α*) independently for different cell types (in this paper, granulocytes).

We will also simulate the stochastic birth-death process for HSCs (see Supplementary Material for details), with the differentiation rate *α* that allows HSCs to differentiate into the progenitor/transit amplifying cell compartment (see Figure 2). Progenitor cells are further distinguished by their generation *ℓ*. Thus, *ℓ* not only measures generation number but also an effective differentiation state. Each HSC differentiation event leads to an *ℓ* = 0 progenitor cell. Since the number of HSCs of any one clone is small, the initial differentiation events from clone *i* follow a Poisson process with rate *αh*_*i*_. The populations of the subsequent generations of progenitor cells in clone *i* are denoted by $n_{i}^{\left(\ell \right)}$ . Once the *ℓ* = 0 generation cells are generated, the number of progenitor cells quickly expand, so their dynamics will be described by a deterministic model as developed in (Xu et al., 2018)

$$\frac{dn_{i}^{\left(\ell \right)}\left(t\right)}{dt}=\left\{\begin{array}{cc} \text{Poisson}\left(\alpha h_{i}\left(t\right)\right)-\left(r_{n}^{\left(0\right)}+\mu_{n}^{\left(0\right)}\right)n_{i}^{\left(0\right)}\left(t\right)\; & \ell =0,\\ 2r_{n}^{\left(\ell -1\right)}n_{i}^{\left(\ell -1\right)}\left(t\right)-\left(r_{n}^{\left(\ell \right)}+\mu_{n}^{\left(\ell \right)}\right)n_{i}^{\left(\ell \right)}\left(t\right)\; & 1\leq \ell \leq L-1,\\ 2r_{n}^{\left(L-1\right)}n_{i}^{\left(L-1\right)}\left(t\right)-\left(\omega +\mu_{n}^{\left(L\right)}\right)n_{i}^{\left(L\right)}\left(t\right)\; & \ell =L, \end{array}\right.$$ (8)

where Poisson(*αh*_*i*_(*t*)) is the time-inhomogeneous point Poisson process describing HSC differentiation events. In other words, after a differentiation event at time *t*_1_, the probability density of the time Δ*t* to the next differentiation event is given by $\alpha h_{i}\left(t_{1}+\Delta t\right)\exp\left[-\alpha \int_{0}^{\Delta t}h_{i}\left(t_{1}+s\right)ds\right]$ . We will use the values of *h*_*i*_(*t*) from our stochastic simulations and sample from this inter-event time density to simulate realizations of differentiation events.

In Eq. 8, $r_{n}^{\left(\ell \right)}$ and $\mu_{n}^{\left(\ell \right)}$ represent the proliferation and death rates of generation-*ℓ* progenitor cells, respectively, and *ω* is the terminal differentiation rate into mature blood. Each division can also be thought of symmetric differentiation producing successively more differentiated progenitor cells. To model the finite proliferative potential of progenitor cells, we set the maximum number of generations to *ℓ* = *L*, after which the *L* ^th^ generation cell can only terminally differentiate to mature blood.

Consider a single isolated differentiation event of an HSC at *t* = 0 belonging to a particular clone. The resulting progenitor cell population after this event is described by Eq. 8 without the Poisson(*αh*_*i*_(*t*)) term but with an initial condition corresponding to a single *ℓ* = 0 cell:

$$n_{i}^{\left(\ell \right)}\left(0\right)=\left\{\begin{array}{cc} 1\; & \ell =0,\\ 0\; & \text{otherwise.} \end{array}\right.$$ (9)

The subsequent populations at time *t* form a temporal “burst” of cells that are described by $n_{i}^{\left(\ell \right)}\left(t\right)$ which is the solution of Eq. 8 without the Poisson(*αh*_*i*_(*t*)) term but using the initial condition in Eq. 9. If we assume that all progenitor generations carry the same division and death rates, $r_{n}^{\left(\ell \right)}=r_{n}$ for 0 ≤ *ℓ* ≤ *L* − 1 and $\mu_{n}^{\left(\ell \right)}=\mu_{n}$ for 0 ≤ *ℓ* ≤ *L*, we can find an analytical solution associated with a single isolated burst as

$$n_{i}^{\left(L\right)}\left(t\right)=\frac{e^{-\left(\omega +\mu_{n}\right)t}}{\left(L-1\right)!}{\left(\frac{2r_{n}}{r_{n}-\omega }\right)}^{L}\int_{0}^{\left(r_{n}-\omega \right)t}z^{L-1}e^{-z}dz.$$ (10)

We can evaluate all populations $n_{i}^{\left(\ell \right)}\left(t\right)$ for *ℓ* < *L* by solving Eq. 8 and using Eq. 10, as detailed in the Supplementary Material.

If we assume that mature cells do not appreciably proliferate^1^, the mature cell population in clone *i* obeys

$$\frac{dm_{i}\left(t\right)}{dt}=\omega n_{i}^{\left(L\right)}\left(t\right)-\mu_{m}m_{i}\left(t\right),$$ (11)

where *μ*_m_ is the lineage-dependent turnover rate of mature cells. Using the solution to $n_{i}^{\left(L\right)}\left(t\right)$ , we solve Eq. 11 to find (see Supplementary Material)

$$m_{i}\left(t\right)=\omega \int_{0}^{t}n_{i}^{\left(L\right)}\left(t^{\prime }\right)e^{-\mu_{m}\left(t-t^{\prime }\right)}dt^{\prime }.$$ (12)

**FIGURE 3:**
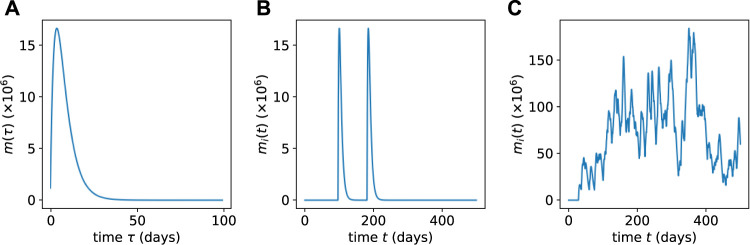
**(A)** The population *m*_*i*_(*t*) of mature cells resulting from a single HSC differentiation event as obtained from Eq. 12. **(B)** Multiple concatenated bursts from a low-population HSC clone showing well-separated intermittent pulses obtained *via* Eq. 13. **(C)** When the HSC population of a clone is large, the resulting mature cell population bursts merge together and exhibit lower relative variability.

The mature cell population burst (of a specific clone) arising from a single, isolated differentiation event is plotted in Figure 3A. Note that the expression for a mature cell burst given in Eq. 12 is derived from the specific initial condition Eq. 9; however, some low-*ℓ* progenitors are also initially transplanted (see below). Thus, *m*_*i*_(*t*) will in general depend on the initial numbers of *n* ^(*ℓ*>0)^(0).

Since Eq. 8 are linear, populations arising from a sequence of Poisson-distributed differentiation events can be constructed by adding those derived from single events occurring at times *T*_*k*_. In this case, the resulting mature cell population at time *t* is given by

$$m_{i}\left(t\right)=\overset{k_{\text{max}}}{\underset{k=1}{\sum }}m_{i}\left(t-T_{k}\right),\;T_{k_{\text{max}}+1}>t>T_{k_{\text{max}}},$$ (13)

where *m*_*i*_(*t* − *T*_*k*_) is the solution given in Eq. 12 (for the specific initial condition in Eq. 9). Two different sequences of bursts are shown in Figures 3B, C. In Figure 3B, we consider a clone with few HSCs such that *αh*_*i*_(*t*) ≪ *μ*_m_. This limit gives rise to differentiation events that occur rarely over the lifespan of mature cells, as depicted Figure 3B. In Figure 3C, we plot a sequence of more frequent mature cell population bursts that arise for more frequent differentiation events *αh*_*i*_(*t*) ≫ *μ*_m_ from HSCs that are in higher population clones.

Recall that the calculations involving Eqs 8–12 are performed for each clone *i*, resulting in a series of time-dependent expressions for *m*_*i*_(*t*) as per Eq. 12. These single event responses are then summed according to Eq. 13 to arrive at the total, time-dependent population of mature cells of a specific type and carrying the same barcode. These predicted whole-organism populations depend on the model parameters $\left\{C_{h}\left(0\right),\alpha ,K,r_{n},\mu_{h},\mu_{n},\mu_{m},L,\omega \right\}$ . Since growth of transit amplifying progenitor cells is fast, we will henceforth assume *r*_n_ ≫ *μ*_n_ ≈ 0. All other rates are given in units of per day.

Since the clone abundances are derived from sequencing cells in a small fraction *η* (for rhesus macaque, *η* ∼ 10^–5^ − 10^–4^) of the animal’s blood taken at times *t*_*j*_, we also need expressions for mature cell populations within a sample. Given the small sample sizes *η*, low population clones in the mature cell pool can easily be missed. For a given population *m*_*i*_ in the whole animal, the probability that *s*_*i*_ cells are captured in the sample is given by (Chao and Lin, 2012; Xu et al., 2020)

Psi|mi,S,M=1MS∏i=0Chmisi≈∏i=0misiηsi1−ηmi−si, (14)

where, for a given cell type (for example granulocytes), *S*(*t*) = *∑*_*i*=0_ *s*_*i*_(*t*) is the total number of sampled cells (including untagged ones), *M*(*t*) = *∑*_*i*=0_ *m*_*i*_(*t*) is the total number of circulating mature cells (including untagged ones), and the sampling fraction is *η* = *S*/*M* (which we first assume is the same at each *t*_*j*_).

After computing *m*_*i*_ at time *t*_*j*_ using Eq. 12, we take the nearest integer value and use it for *m*_*i*_ in Eq. 14. We then draw a single value *s*_*i*_(*t*_*j*_) from the binomial distribution, assuming *η* is given. Finally, we simulate our model to generate trajectories of *M*(*t*) and then determine the tagged sampled fraction

$$S\left(t\right)\approx \frac{H^{*}}{H}\eta M\left(t\right).$$ (15)

Equations 5–15 represent a hybrid stochastic-deterministic model since the self-renewal process of a small number of HSCs in each clone and the final sampling step (Eq. 14) are modeled as discrete stochastic processes, while proliferation and differentiation of higher population progenitor and mature cells are treated deterministically *via* Eqs 8–12. The values *s*_*i*_(*t*_*j*_) obtained from the model in Eqs 8–14 are used to generate the predicted clone population mean and standard deviations, *s*_*i*_ and *σ*_*i*_, according to Eq. 1. Both *s*_*i*_, *σ*_*i*_ are then used to determine the density of points *ρ*_*i*_. These three values for each clone are then compared to their corresponding values constructed from data ${\hat{s}}_{i}\left(t_{j}\right)$ , as we detail in the next section. These predictions, along with the predicted richness *C*_s_(*t*) and total sampled granulocyte population *S*(*t*) provide the basis for comparing with measured data and parameter inference.

## Data analysis and results

### Data presentation

The published experimental data in (Koelle et al., 2017) provide data for four rhesus macaques ZH33, ZG66, ZH19, and ZJ31 over a period of 49, 42, 36 and 20 months, respectively. Samples were taken after autologous transplantation with myeloablative conditioning at times *t*_*j*_, 1 ≤ *j* ≤ *J*. Possible sampled cells are of five types: T cells, B cells, monocytes, granulocytes and NK cells. Barcoded myeloid (granulocytes and monocytes) and B-cells reach a noisy equilibrium after approximately 1 month, whereas for T-cells the time frame is longer, between 5 to 17 months. Furthermore, since granulocytes comprise a majority of white blood cells, we apply our mathematical and statistical model to the granulocyte lineage. At each sampling time *t*_*j*_, the experimental data from (Koelle et al., 2017) reveals how many cells are sampled from each clone and what type each cell is. Across sampling time points, these sampled populations contain information on the overall abundance, how these abundances fluctuate in time, and the density of the number of clones detected as a function of abundance and abundance variability. Since ZH33 has the longest follow-up period, we use data from this macaque to compare experimental data with our mathematical predictions.

### Matching model to data

Validation of our model will rely on matching predictions with available data in the form of ${\hat{C}}_{s}\left(t_{j}\right)$ , $\hat{S}\left(t_{j}\right)$ , and $\left({\hat{s}}_{i},{\hat{\sigma }}_{i},\hat{\rho }\left(s,\sigma \right)\right)$ . Since the model contains many parameters and the data is noisy and “sparse,” the model will likely overfit. Therefore, we carry out the parameter estimation by hand in stages, imposing limit on parameter values that are physiologically feasible.

First, we compare *C*_h_(*t*) (Eq. 6) with the richness ${\hat{C}}_{s}\left(t_{j}\right)$ shown in Figure 1A to provide a constraint among *μ*_h_, *K*, *C*_h_(0), and *r*_h_(0). We assume that the *C*_h_(0) associated with granulocytes is slightly greater than the total richness across all samples after the first two, $C_{h}\left(0\right)≳{\hat{C}}_{s}^{>2}$ . This is equivalent to assuming that granulocyte richness after about 2 months arises solely from barcoded HSCs. The cumulative post-two-month richnesses ${\hat{C}}_{s}^{>2}$ for animals ZH33, ZG66, ZH19, and ZJ31 are 2,335, 2007, 4,007, and 30732, respectively. Although the sample specific ${\hat{C}}_{s}\left(t_{j}\right)$ quickly decreases for *t* > *t*_1_, our model prediction for *C*_h_(*t*) follows Eq. 6 and decays more slowly. By estimating *C*_h_(0) and using Eq. 6, we generate the predicted *C*_s_(*t*) and *S*(*t*) by simulating our full model and comparing them to ${\hat{C}}_{s}\left(t_{j}\right)$ and $\hat{S}\left(t_{j}\right)$ . This allows us to further constrain the parameters *C*_h_(0), *r*_h_(0), *K*, and *μ*_h_. Note that no analytic formula exists for *C*_s_(*t*).

We first set *K* ≈ 100*C*_h_(0) as the niche carrying capacity since smaller or larger values of *K* cannot provide the correct average clone sizes or approximately matching values of *C*_s_. This comparison allowed us to obtain rough constraints and approximations to some parameters, particularly *μ*_h_, *r*_h_(0), and *C*_h_(0). Discrete sets of values that are consistent with ${\hat{C}}_{s}\left(t_{j}\right)$ were selected and further pruned by using the remaining data.

The sampled richness ${\hat{C}}_{s}\left(t_{j}\right)$ in ZH33 exhibits a sharp decrease after the first sample time, without a corresponding collapse in the sampled abundances of granulocytes $\hat{S}\left(t_{j}\right)$ . Our model explains this phenomenon by the initial condition; namely, the initially transplanted population of CD34^+^ cells contains some partially differentiated HSPCs. Progenitor cells of barcode *i* (described in our model by the populations $n_{i}^{\left(\ell \right)}$ ) are initially transplanted so that some $n_{i}^{\left(\ell \right)}\left(t=0\right)>0$ particularly for small *ℓ* (cells with a low degree of differentiation).

**FIGURE 4:**
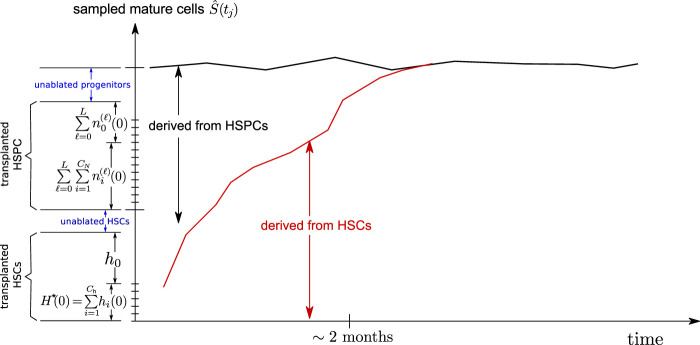
Schematic of a mixed HSC/HSPC initial condition. The transplanted CD34^+^ cells contain HSCs and some progenitor cells (HSPCs), which are exhausted after about 2 months. The remaining richness arises mainly from that of the long term HSCs *C*_h_(0) which then slowly decreases as certain HSC clones become extinct.

As shown in Figure 4, a fraction of the initial clones are HSPCs. Once these HSPC clones generate a burst of mature cells, they disappear from the animal without being renewed since there are no corresponding HSCs carrying the same barcode. Thus, the HSPC contribution to the overall sampled richness ${\hat{C}}_{s}\left(t_{j}\right)$ largely disappears after about 2 months. However, the total mature granulocyte population $\hat{S}\left(t_{j}\right)$ does not suffer a decline since HSPCs lost due to terminal differentiation are replaced by HSC differentiation. The subsequently sampled mature cell richness then reflects the richness *C*_h_(0) of the initially transplanted HSCs. We propose this partial HSPC transplantation as a mechanism for the observed rapid decrease in ${\hat{C}}_{s}$ observed in some animals. The shape of $\hat{\rho }\left(s\right)$ (see Figure 7D) can inform our estimate of the initial progenitor population *n* ^(*ℓ*)^(*t* = 0). Maxima in $\hat{\rho }\left(s\right)$ can be accounted for by offspring of initially transplanted progenitor cells of different stages *ℓ*, with *n* ^(*ℓ*)^(0) generating smaller clones for larger *ℓ* (fewer remaining generations to expand).

Next, we consider the small clones, predominantly arising at short times, and find their average value at the first sample taken at *t*_1_. These small clones also yield the highest density values $\hat{\rho }\left(s\right)$ , but mostly disappear at longer times. Therefore, we assume they predominantly arise from initial progenitor cells. We can then generate the prediction *m*(*t* = *t*_1_) from our model assuming an initial condition *n* ^*ℓ*^(0) (and assuming no HSC contribution by setting *α* = 0). This approximation provides a constraint on the deterministic progenitor cell parameters *r*_n_, *L*, *ω*, *η*. In these experiments, the typical *η* ∼ 10^–5^, so we find that *L* = 22, and collect a set of feasible values for *r*_n_, *ω*, and *η* that provide a good starting point for estimating the other parameters in the model. Note that *r*_n_, *L*, *ω*, *η* can largely compensate each other at this level of comparison. In other words, sets of different ranges of values of one parameter will fit equally well provided some other parameters are also correspondingly adjusted.

**FIGURE 5:**
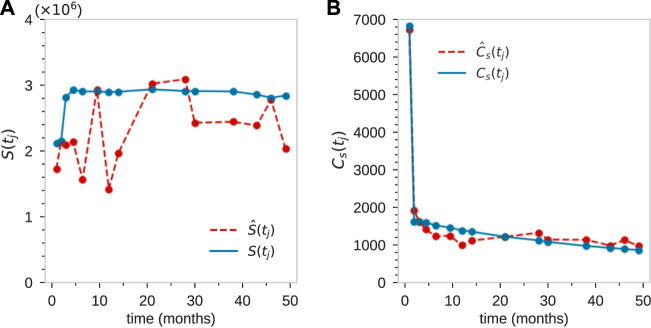
For animal ZH33, coarse fitting to the total population of tagged granulocytes *S*(*t*_*j*_) **(A)**, and the total number of clones at each time point *C*_s_(*t*_*j*_) **(B)**. This initial rough matching was achieved using parameter values *L* = 22, *r*_n_ ≈ 2, *ω* ≈ 0.2, and sampling size *η* = 10^–5^.

Next, we least-squares minimize $\hat{S}\left(t_{j}\right)-S\left(t_{j}\right)$ , where the model prediction *S*(*t*) is given by Eq. 15. This further helps fix *α*. Once a set of parameters that allow for a reasonable match of *C*_s_(*t*_*j*_) and *S*(*t*_*j*_) have been defined, as shown in Figures 5A, B, we then refine the fitting to the mean−standard deviation $\left({\hat{s}}_{i},{\hat{\sigma }}_{i}\right)$ scatter plot shown in Figure 1D.

To compare our modeling results to the remaining experimental data ( ${\hat{s}}_{i},{\hat{\sigma }}_{i}$ , and ${\hat{\rho }}_{i}$ ) and to fine-tune the estimates of the other parameters, the most natural intuition would be to compute the Euclidean distance (or any other relevant distance metric) between model predictions and experimental datasets and tune parameters so as to minimize this distance. However, since a number of clones go extinct in our model, the data size (for ${\hat{s}}_{i},{\hat{\sigma }}_{i}$ , and ${\hat{\rho }}_{i}$ ) varies in time.

Thus, before comparing predicted clone size distributions to measured results, we first cluster the data according to the values of ${\hat{s}}_{i}$ and ${\hat{\sigma }}_{i}$ . Recall that $\hat{\rho }\left(s,\sigma \right)\approx \hat{\rho }\left(s\right)$ (since *σ* is highly correlated with *s*) is still determined from KDE using the raw, unclustered data $\left({\hat{s}}_{i},{\hat{\sigma }}_{i}\right)$ . Clustering is performed using *k*-means to partition the data into multiple regions (in $\hat{s}$ - and $\hat{\sigma }$ -space) such that the Euclidean distance between a point and the center of its cluster is smaller than its distance to all other cluster centers. The goal is not to cluster the $\left({\hat{s}}_{i},{\hat{\sigma }}_{i}\right)$ points according to any real feature, but to simply reduce the dimensionality of the problem and to control the number of effective data points before applying least squares comparisons. Although there are no obvious features in the $\left({\hat{s}}_{i},{\hat{\sigma }}_{i}\right)$ data, *k*-means clustering of the distribution of points does yield an optimal number of clusters k* *via* the “elbow” method where the curvature of the sum of square errors (distortion score) is maximal (Yuan and Yang, 2019). After implementing *k*-means clustering using the Python *yellowbrick* package, we find that the optimal number of clusters is typically k* ≈ 50 ± 3 depending on the initial randomization and partitioning process. Subsequent results, however, are insensitive to the precise numbers of clusters used as long as k* ≈ 50.

**FIGURE 6:**
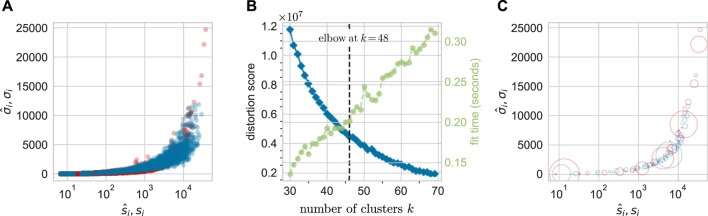
A total of 48 clusters are identified *via* *k*-means clustering of the data from animal ZH33. **(A)** The unclustered $\left({\hat{s}}_{i},{\hat{\sigma }}_{i}\right)$ data and model predictions for an arbitrary set of parameters. **(B)** The squared errors in *k*-means clustering or distortion score (blue) and convergence time (green) as a function of the number of clusters. The optimal value from the elbow signalling diminishing returns was found in this case to be k* = 48. **(C)** The location of the cluster centers plotted on the $\left(\hat{s},\hat{\sigma }\right)$ plane. The radius of each circle indicates the fraction of all clones *w*_*k*_ that are associated with cluster *k* and is set to 2000*w*_*k*_.

Figure 6A compares $\left({\hat{s}}_{i},{\hat{\sigma }}_{i}\right)$ from experiments and from our hybrid multicompartment model. Figure 6B shows the distortion score (blue) and the convergence time (green) as a function of the number of clusters. The optimal number of clusters k* = 48 arises at the elbow of the distortion score curve. Figure 6C shows the clustered data (for animal ZH33) against the clustered model predictions. The radius of each circle, *w*_*k*_, *k* = 1, …, 53 denotes the fraction of data points (fraction of the total number of observed clones) assigned to cluster *k* and is thus a coarse-grained representation of the local data density.

Thus, we have clustered measured data according to $P=\left\{p_{1},\ldots ,p_{k^{*}}\right\}\equiv \left\{\left({\hat{s}}_{1},{\hat{\sigma }}_{1},{\hat{w}}_{1}\right),\ldots ,\left({\hat{s}}_{k^{*}},{\hat{\sigma }}_{k^{*}},{\hat{w}}_{k^{*}}\right)\right\}$ , where $\left({\hat{s}}_{k},{\hat{\sigma }}_{k}\right)$ denotes the center values of cluster *k* and ${\hat{w}}_{k}$ is the area of the *k* ^th^ cluster. For a fixed set of all model parameters, we generate predictions $Q=\left\{q_{1},\ldots ,q_{g^{*}}\right\}\equiv \left\{\left(s_{1},\sigma_{1},w_{1}\right),\ldots ,\left(s_{\ell^{*}},\sigma_{\ell^{*}},w_{\ell^{*}}\right)\right\}$ (in this expression and in the following, *ℓ*, ℓ* denote matrix indices and not progenitor cell generations). The optimal number of clusters derived from our hybrid stochastic-deterministic model, ℓ* will in general be different from the number of clusters k* derived from data, but is typically also about ℓ* ≈ 50.

In order to compare the clustered data *P* to the model prediction *Q*, which are matrices with different numbers of rows, we use the Wasserstein metric, or Earth mover’s distance (EMD) (Villani, 2009; Kolouri et al., 2017) to define a distance between them. Let *d*_*k*,*ℓ*_ denote the distance between cluster *p*_*k*_ and cluster *q*_*ℓ*_ so that the matrix a **d** = {*d*_*k*,*ℓ*_} catalogues all possible cluster-cluster distances. We aim to compute a flow map **f** = {*f*_*k*,*ℓ*_} that yields the minimal distance between clusters **p** = {*p*_*k*_} and clusters **q** = {*q*_*ℓ*_} by finding

$${\text{min}}_{f}\overset{k^{*}}{\underset{k=1}{\sum }}\overset{\ell^{*}}{\underset{\ell =1}{\sum }}f_{k,\ell }d_{k,\ell }$$ (16)

Subject to the constraints

$$f_{k,\ell }\geq 0,\;\overset{k^{*}}{\underset{\ell =1}{\sum }}f_{k,\ell }\leq w_{k},\;\overset{\ell^{*}}{\underset{k=1}{\sum }}f_{k,\ell }\leq w_{\ell },$$ (17)

For all 1 ≤ *k* ≤ k* and 1 ≤ *ℓ* ≤ ℓ* and

$$\overset{k^{*}}{\underset{k=1}{\sum }}\overset{\ell^{*}}{\underset{\ell =1}{\sum }}f_{k,\ell }=\overset{k^{*}}{\underset{k=1}{\sum }}w_{k}=\overset{\ell^{*}}{\underset{\ell =1}{\sum }}w_{\ell }.$$ (18)

After finding the optimal flow **f**, we evaluate the EMD as

$$EMD\left(P,Q\right)=\frac{\overset{k^{*}}{\underset{k=1}{\sum }}\overset{\ell }{\underset{\ell =1}{\sum }}f_{k,\ell }d_{k,\ell }}{\overset{k^{*}}{\underset{k^{\prime }=1}{\sum }}\overset{\ell^{*}}{\underset{\ell^{\prime }=1}{\sum }}f_{k^{\prime },\ell^{\prime }}}.$$ (19)

Model parameters are varied until our model-derived predictions best match the clustered data by minimizing the EMD. In this paper, we consider only the granulocyte lineage since it is the most abundant and reliably measured with minimal complex dynamics and regulation.

**FIGURE 7:**
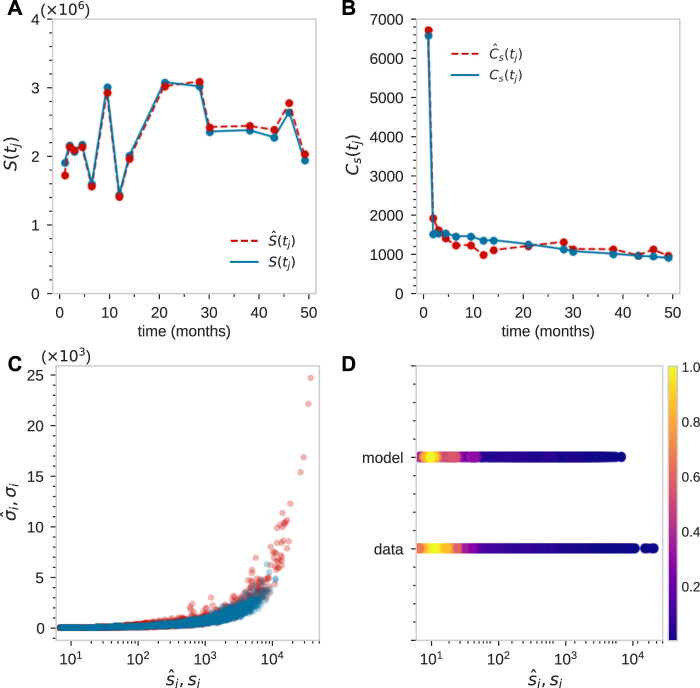
Fitting of tagged granulocyte populations for animal ZH33 after time-dependent adjustment of sampling size *η*(*t*_*j*_). For this animal *t*_*j*_ = [1, 2, 3, 4.5, 6.5, 9.5, 12, 14, 21, 28, 30, 38, 43, 46, 49] months. **(A)** By initially best-matching *S*(*t*_*j*_), we find *η*(*t*_*j*_) = [1, 2.03, 1.07, 0.94, 0.68, 1.25, 0.61, 0.83, 1.29, 1.29, 1.01, 1.01, 0.99, 1.14, 0.83]×10^–5^. **(B–D)** By searching parameter space to find a good match to ${\hat{C}}_{s}\left(t_{j}\right),{\hat{s}}_{i},{\hat{\sigma }}_{i}$ , and $\hat{\rho }$ (which is shown with its maximum nnormalized to one), which is plotted with its maximum normalized to unity. We find parameters [in units of/day] that fit the data are *μ*_h_ = 0.02, *r*_h_(0) = 0.08, *C*_h_(0) = 2500, *C*_n_(*ℓ* = 0) = 800, *C*_n_(*ℓ* = 1) = 1600, *C*_n_(*ℓ* = 2) = 3200, *K* = 2.5×10^5^, *α* = 0.016, *r*_n_ = 2, *L* = 22, *μ*_n_ = 0, *μ*_m_ = 0.185 and *ω* = 0.2.

Finally, note the fluctuations in *S*(*t*_*j*_) which are too large to be captured by the intrinsic stochasticity in our model, as indicated in Figure 5B. These “unknown” fluctuations can arise from a number of processes, including variable sampling fractions *η*(*t*_*j*_) at each time point *t*_*j*_ and fluctuating animal state due to infections, stress, inflammation, etc. These may influence total mature cell populations month to month. Some of these effects can be effectively accounted for by adjusting the mean sample fraction *η* = 10^–5^ by an amount Δ*η*(*t*_*j*_) at each time point. Using these values of Δ*η*(*t*_*j*_) to match the data *S*(*t*_*j*_), and then readjusting the parameters, we find good comparison between the experimental measurements and our model, as shown in Figures 7A–C.

To further show consistency, we then plotted the predicted density and compare it with the data-derived (using KDE) density $\hat{\rho }$ in Figure 7D. A few large, highly variable clones remain not well reproduced by our model and are discussed in the next section. The parameter estimation procedure was applied to the three other animals in (Koelle et al., 2017), showing reasonable, consistent matching (see Supplementary Material).

## Discussion and conclusion

In this paper, we analyzed data from stem cell transplantation experiments in rhesus macaque (Koelle et al., 2017) in which barcoded stem and progenitor cells (HSPCs) were autologously transplanted after myeloablative conditioning. Typically, of the ∼30 million cells transplanted only ∼15–35% are tagged and then only a fraction homes to functional bone marrow niches. Nonetheless, typically hundreds or thousands of barcodes are detected in peripheral blood samples. The counts of circulating mature cells derived from each clone fluctuate from sample to sample; these fluctuations are significantly larger than those expected from random small samples (Xu et al., 2018) and thus arise from intrinsic stochasticity (Abkowitz et al., 1996) during hematopoiesis and/or physiological changes in the animals over the months that samples were being drawn.

In order to explain clone size variability, we extend a mathematical model first presented in (Xu et al., 2018). Our hybrid stochastic-deterministic model delineates all the clone populations and assumes regulation of the stem cell proliferation rate through a carrying capacity *K*, a finite differentiation potential of progenitor cells, terminal differentiation after a fixed number of divisions, and a final sampling step. Since the numbers of HSCs within each clone are typically small, we treated the self-renewal of HSCs within its niche stochastically by a coupled (through the carrying capacity) discrete birth-death process for each clone. In (Xu et al., 2018), the coupling in the stochastic HSC birth-death process was treated using a mean field approximation, which leads to a smaller clone size variability, everything else equal. Random times of asymmetric differentiation of each HSC into the first-stage progenitor cell is described by a rate *α*. After *L* differentiation steps, the *L* ^th^-stage progenitor cell terminally differentiates into a mature, circulating blood cell. The progenitor and mature cell pools are treated deterministically. We performed stochastic simulations using the Gillespie algorithm (Bortz et al., 1975; Gillespie, 2007) of the entire HSC pool and solve for the progenitor and mature cell populations numerically. Additional feedback mechanisms between the HSC and progenitor pools can be implemented (Klose et al., 2019), but in our case would require a more complex model incorporating two-way coupling between stochastic and deterministic dynamics.

Our model suggests that the sampled clone abundance variation arises primarily from random differentiation events by HSC clones that occur at rate *α*. Each differentiation event leads to a temporal burst of mature cells of the same clone. We hypothesize that these bursts of mature cell production lead to the variability in sampled clone populations. Other physiological factors related to animal state may still also play a role, but are not considered in our model. Another feature captured by our current model is the transient richness immediately after transplantation. This behavior is explained by our initial condition that contains short-term HSPCs that are within the initial CD34^+^ pool. These HSPCs quickly differentiate but are not replenished at long times. This distributed initial condition may depend on the experimental protocol and may be indicative of the efficiency, and especially, the composition of the transplant.

Our model also allows us to explore how clone abundance predictions change with parameters. For example, we find that the range of larger clone sizes increase with increases in *L*, *K*, and *α*, in this order. All parameters affect the density of predicted data and cluster sizes. Mature cell death rates, which vary across different cell types, also affect the predicted abundance variations, especially for larger values of *μ*_m_.

We also note that while percentage of GFP+ cells within each mature cell type (lineage) fluctuated from sample to sample, they generally increased with animal age (after transplant) for all animals. T cells had the largest increase in their barcoded populations during the handful of months after transplantation. Since this timescale is much longer than the progenitor cell transients, this slow increase in tagged cell populations suggests that, assuming a neutral model (barcodes and barcode integration sites do not affect cell proliferation and death rates), (i) that a certain fraction of HSCs remained *in situ* after radiative ablation, and (ii) the CD34^+^ barcoded and transplanted HSC population with an enriched GFP+ fraction were slowly activated. Scenario (i) means that some HSCs remained in their niche and continuously generated blood. The transplanted cells with barcoded HSCs (GFP+) can increase in importance if they slowly become more proliferative as they settle into the animal. Thus, the slow increase in GFP+ fraction in all five measured lineages suggest that transplanted cells may recover slowly from the transplantation procedure and increase their contribution to mature blood cell formation.

Finally, we note that there appears to be additional fluctuation mechanisms that are not accounted for in our model. In all animals, there appears to be a few very large clones with very high variability ${\hat{\sigma }}_{i}$ . Within a stochastic HSC population, adjusting birth-death parameters that allow for larger clones and larger variances would suppress the richness to below what is observed. We have extensively explored feasible regimes of all parameters and conclude that allowing large clones that vary in abundance precludes agreement with other basic observables such as $\hat{S}\left(t_{j}\right)$ and ${\hat{C}}_{s}\left(t_{j}\right)$ . Nonetheless, such unexplained features can be mechanistically informative and we discuss a number of reasonable factors that may account for them. First, the fluctuations in the measured total abundances of the different mature cell lineages did not correlate, implying that the specific set of sampling sizes *η*(*t*_*j*_) used to explain granulocyte population variations (as shown in Figure 7) cannot be used to analyze those of other mature cell lineages. The seemingly uncorrelated cross-lineage populations imply that time variations in animal state arise further downstream, affecting the development of individual lineages. If fluctuations occurred in stem or multipotent progenitor cells, they would affect multiple cell lineages in similar ways and lead to inter-lineage population correlations.

Animal ZJ31 (see Supplementary Material) appears to be uniquely different from the others in that it exhibited a much larger richness ${\hat{C}}_{s}\left(t_{j}\right)$ as well as a much smaller maximum clone size (which non-etheless had high variance ${\hat{\sigma }}_{i}$ ). For example, *C*_s_ at *t*_*j*_ = 5 months dips to a very low value, while the abundance $\hat{S}\left(t_{j}\right)$ seems to be at a local maximum. Thus, a small number of granulocyte clones expanded dramatically, potentially squeezing out the many smaller clones below sampling. At month seven, $\hat{S}$ is extremely low but ${\hat{C}}_{s}$ has recovered to its long term value, indicating that the previously large granulocyte clones were quickly cleared out. The results for ZJ31 may indicate a lower level of competitive exclusion, but also some other mechanism contributing to high variability. Therefore, the overall observation of high variability and the magnitudes of ${\hat{C}}_{s}$ indicates that other model features should be considered.

One assumption of our model that is likely an oversimplification is the neutrality of barcodes. Although different barcodes themselves may not influence cells, different VISs may. For example, aberrant self-renewal arises when using lentiviral vectors (Espinoza et al., 2019) and different VISs of HIV have been shown to affect cellular proliferation rates (*e.g.*, if the VIS is near an oncogene) Yeh et al. (2021). Besides the non-neutrality, we have also neglected stochastic or variable proliferative potential *L* and the time course of the HSC homing and engraftment into the bone marrow. A random but distributed *L* would allow a few randomly selected clones to expand further. We also expect that HSC migration and successful settlement into the bone marrow niche is a time-continuous process that provide a proliferation head start for a few early arriving clones. This would ultimately result in fewer clones *C*_s_ with some of them at higher populations *s*_*i*_(*t*). An instantaneous (more abrupt) HSC engraftment and a larger spread in *L* would be more consistent with animal ZJ31 than with the others.

Additional information can be extracted from the clone abundance data to further identify and interrogate such “opposing” behaviors. For example, we have only considered the average autocorrelation of the clone abundances (the variance) and have not constructed cross-correlations between cell types/lineages or correlations across time. Except for the initial period after transplantation, we have assumed a time-inhomogeneous process and have not considered explicit time-dependence such as aging (Muller-Sieburg et al., 2012; de Haan and Lazare, 2018). Physiological aging can be straightforwardly incorporated by *e.g.,* allowing for slow degradation of HSCs, changes in progenitor proliferative potential (Marciniak-Czochra et al., 2009), or changes in HSC niche carrying capacity *K*(*t*). Mutations that arise with age may also *increase* HSC self-renewal (Challen and Goodell, 2020) which could be modeled by a *r*_h_(0) that increases with time. Thymic interruptions or involution with age (Lewkiewicz et al., 2019a,b) could also be modeled by assuming a decreasing maturation rate *ω*(*t*) when considering the T cell lineage.

While our current model contains a large number of parameters, it seems that a number of them are compensatory and control specific properties of the model predictions. For example, we found that *α*, *L*, *ω*, and *η* can compensate for each other and form an unknown effective parameter function *f*(*α*, *L*, *ω*, *η*). This feature effectively reduces overfitting and might be better analyzed using machine learning methods. Incorporating the more realistic mechanisms discussed above would yield additional effective parameters allowing the model to more accurately reproduce the measured quantities; nonetheless, intermittent differentiation of HSCs remains the key proposed mechanism for understanding intersample clone abundance variations.

## Data Availability

Publicly available datasets were analyzed in this study. This data can be found here: https://doi.org/10.1182/blood-2016-07-728691.

## Footnotes

1 Certain lineages such as T cells can intermittently proliferate, e.g., upon antigen activation, but we neglect this and use small effective death rates *μ*_m_ for such cell types.
