# Supplementary material for: Clonal abundance patterns in hematopoiesis: Mathematical modeling and parameter estimation
Source: Front Syst Biol. 2023 Feb 9;3:893366. doi: 10.3389/fsysb.2023.893366 (PMC12342013; doi:10.3389/fsysb.2023.893366)
Supplement: Supplementary file 1 [file DataSheet1.pdf]

SUPPLEMENTARY MATERIAL

A. Stochastic simulation procedure

Our stochastic hematopoiesis simulations are based on the clone-resolved mechanistic model presented in the main text and using initial conditions and parameters consistent with data obtained from the rhesus macaque ZH33 from (Koelle et al., 2017). We simulate the dynamics in the HSC population in which individual cells are subject to self-renewal at rate $r_{\rm h}(h(t))$ given by Eq. 5, death at constant rate $\mu_{\rm h}$, and differentiation into progenitor cells of type i through a Poisson process with rate $\alpha h_i(t)$. Progenitor cells are assumed to evolve deterministically according to Eqs. 8. We then apply the analytical solution in Eq. 10 (see Appendix B) to obtain the L-generation progenitor cell count $n_i^L(t)$ and subsequently the mature cell count $m_i(t)$ in each clone at time t. Finally, we sample via the binomial distribution in Eq. 14 to obtain a sampled mature cell count $s_i(t_j)$ for each clone at each sampling time t_j. The specific steps are

Step 1. Set the initial conditions. Start with $C_{\rm h}(0)$ clones and initial conditions $h_i(0) = 1$, $n_i^{(\ell)}(0) = 0$ (for $0 \leq \ell \leq L$), and $m_i(0) = 0$, for $1 \leq i \leq C_{\rm h}$. This implies that $h(0) = C_{\rm h}(0) + h_0(0)$ since in the definition of $h(t)$ we also include untagged cells $h_0(0)$. Since we used $C_{\rm h}(0) \approx 2500$, and $\sim 65\%$ of sampled cells are untagged, we take $h_0(0) \approx 4700$. In practice, we only explicitly consider differentiation of tagged cells ($i = 1, \ldots, C_{\rm h}$) and assume that the fraction of untagged cells propagates through to the sampled cells. For each clone, we also create a list of differentiation event times T_k corresponding to the instances when zeroth-generation progenitor cells are created. The list is updated every time a new generation event occurs.

Step 2. The HSC dynamics in each each clone i follows a Poisson process of self-renewal, death and differentiation according to the rates $r_{\rm h} h_i(t)$, $\mu_{\rm h} h_i(t)$ and $\alpha h_i(t)$, respectively. A Gillespie algorithm is used to simulate the Poisson process (Gillespie, 1977), according to Steps 3-5. The total number of HSCs $h(t) = \sum_{i=0}^{C_{\rm h}} h_i(t)$ is updated whenever its number changes through self-renewal or death.

Step 3. For each clone, randomly select a random number $u_1^{(i)}$ from the uniform distribution $[0, 1]$. to determine the time of occurrence of the next event, which may be either self-renewal, death or differentiation of the HSC cells. We assume we are at time t and that events are generated via an overall Poisson process of rate $\lambda_i = (r_{\rm h}(h(t)) + \mu_{\rm h} + \alpha)h_i(t)$. The Poisson likelihood that q events will occur within time $\Delta t^{(i)}$ is given by $P(X = q) = (\lambda_i t)^q e^{-\lambda_i \Delta t^{(i)}}/q!$ so that the probability $P(T_1 \leq \Delta t^{(i)})$ that the first event will occur within the inter-arrival time $\Delta t^{(i)}$ is

$$P(T_1 \leq \Delta t) = 1 - P(X = 0) = 1 - e^{-\lambda_i \Delta t^{(i)}} = u_1^{(i)}. \tag{20}$$

Interarrival times are then calculated from $\Delta t^{(i)} = -\ln(1 - u_1^{(i)})/\lambda_i$.

Step 4. Since there are i clones, each of them will be associated with an interarrival time $\Delta t^{(i)}$. We select the smallest among them, $\Delta t^{(i_*)} = \min_i \{\Delta t^{(i)}\}$ and focus on the corresponding clone i_*. Time is shifted to $t \to t + \Delta t^{(i_*)}$.

Step 5. To determine whether the next event in clone i_* is self-renewal, death or differentiation we draw a new random number u_2 within the uniform range $[0, 1]$ and use it to determine the likelihood of self-renewal $P_{\rm r}(t)$ and death $P_\mu(t)$. These are given as $P_r = r_{\rm h}(h(t))(r_{\rm n}(h(t)) + \mu_{\rm h} + \alpha)^{-1}$ and $P_\mu = \mu_{\rm h}(r_{\rm n}(h(t)) + \mu_{\rm h} + \alpha)^{-1}$, respectively. If $u_2 < P_r$, a new HSC is generated, if $P_r \leq u_2 < P_r + P_\mu$, an HSC will be removed. Otherwise for $u_2 \geq P_r + P_\mu$, the HSC will differentiate into a zero-generation ($\ell = 0$) progenitor cell.

Step 6. If self-renewal is selected we also increase the value of $h(t)$ by one. If differentiation is selected we add the time of the new $\ell = 0$ generation event for clone i^* in the list.

Step 7. Repeat the same procedure in Steps 3 to 6 until a final cutoff time $t_{\max}$.

Step 8. For each HSC clone, associate the finalized list of differentiation (generation of a new $\ell = 0$ progenitor cell) event times with T_k in Eq. 13.

Step 9. Evaluate the number of mature cells $m_i(t = t_j)$ of a given type at the sampling times $t = t_j$ for $1 \leq j \leq J$. Take the nearest integer m_i and determine the number of sampled cells $s_i(t_j)$ by drawing one realization from the probability distribution in Eq. 14 at each time point t_j and use these values in Eqs. 1 to find predicted population means and variances. These predicted values are then compared with experimental values $\{\hat{s}_i, \hat{\sigma}_i\}$ for $1 \leq i \leq C_{\rm s}(\text{cell type})$ from animal ZH33.

B. Analytic expressions for unit differentiation burst

Here, we find the analytical solution to the deterministic model presented in Eqs. 8 for the populations $n^{(\ell)}(\tau)$ of any given clone at time τ after a single differentiation event that occurred at time zero. For a single event, Eqs. 8 can be written for times $\tau > 0$ *without* the influx term

$$\frac{\mathrm{d}n^{(\ell)}(\tau)}{\mathrm{d}\tau} = \begin{cases} -\left(r_{\rm n}^{(0)} + \mu_{\rm n}^{(0)}\right) n^{(0)}(\tau) & \ell = 0, \\ 2r_{\rm n}^{(\ell-1)} n^{(\ell-1)}(\tau) - \left(r_{\rm n}^{(\ell)} + \mu_{\rm n}^{(\ell)}\right) n^{(\ell)}(\tau) & 1 \leq \ell \leq L-1, \\ 2r_{\rm n}^{(L-1)} n^{(L-1)}(\tau) - \left(\omega + \mu_{\rm n}^{(L)}\right) n^{(L)}(\tau) & \ell = L, \end{cases} \tag{21}$$

but with an initial condition

$$n^{(\ell)}(0) = \begin{cases} 1 & \ell = 0 \\ 0 & \text{otherwise} \end{cases} \tag{22}$$

to describe the dynamics immediately following a single differentiation event.

For simplicity we assume constant $r_{\rm n}^{(\ell)} = r_{\rm n}$ and $\mu_{\rm n}^{(\ell)} = \mu_{\rm n}$ are independent of generation number ℓ; the terminal differentiation step may carry a different rate ω. Since the above equations are linear we can use the Laplace transform

$$\mathcal{L}[n^{(\ell)}(\tau)] \equiv n^{(\ell)}(s) = \int_0^\infty n^{(\ell)}(\tau) e^{-s\tau} \mathrm{d}\tau \tag{23}$$

and the initial condition $n^{(0)}(s) = 1/s$ to convert Eqs. 21 to algebraic equations, yielding

$$n^{(\ell)}(s) = \frac{(2r_{\rm n})^\ell}{(s + r_{\rm n} + \mu_{\rm n})^{(\ell+1)}} \quad \text{and} \quad n^{(L)}(s) = \frac{(2r_{\rm n})^L}{(s + r_{\rm n} + \mu_n)^L (s + \omega + \mu_{\rm n})}. \tag{24}$$

These can then be inverted using the identities

$$\mathcal{L}^{-1}\left(\frac{1}{(s+a)^{n+1}}\right) = \frac{\tau^n e^{-a\tau}}{n!} \quad \text{and} \quad \mathcal{L}^{-1}\left(f(s)g(s)\right) = \int_0^\tau f(\tau - t')g(t')\mathrm{d}t'$$

to find

$$
\begin{aligned}
n^{(0)}(\tau) &= e^{-(r_{\rm n}+\mu_{\rm n})\tau} \\
n^{(\ell)}(\tau) &= \frac{(2r_{\rm n})^{\ell}}{\ell!}\tau^{\ell}e^{-(r_{\rm n}+\mu_{\rm n})\tau} \\
n^{(L)}(\tau) &= \frac{e^{-(\omega+\mu_{\rm n})\tau}}{(L-1)!}\left(\frac{2r_{\rm n}}{r_{\rm n}-\omega}\right)^{L}\int_{0}^{(r_{\rm n}-\omega)\tau} z^{L-1}e^{-z}{\rm d}z.
\end{aligned}
\tag{25}
$$

Finally, the expression for $n^{(L)}(\tau)$ can be used to determine the number of mature cells $m(\tau)$ via

$$
\frac{{\rm d}m(\tau)}{{\rm d}\tau} = \omega n^{(L)}(\tau) - \mu_{\rm m}m(\tau), \tag{26}
$$

which is solved to find

$$
m(\tau) = \omega\int_{0}^{\tau} n^{(L)}(\tau')e^{-\mu_{\rm m}(\tau-\tau')}{\rm d}\tau'. \tag{27}
$$

For each clone i, responses corresponding to Poisson-distributed differentiation events are summed according to Eq. 13 to arrive at the final time course of the mature cell population $m_i(t)$. Note that $m_i(t)$ in Eq. 27 is derived under the simple initial condition in Eq. 22. Different initial conditions $n^{(\ell)}(0)$ would yield different forms for $m_i(t)$.

C. Fitting for all animals

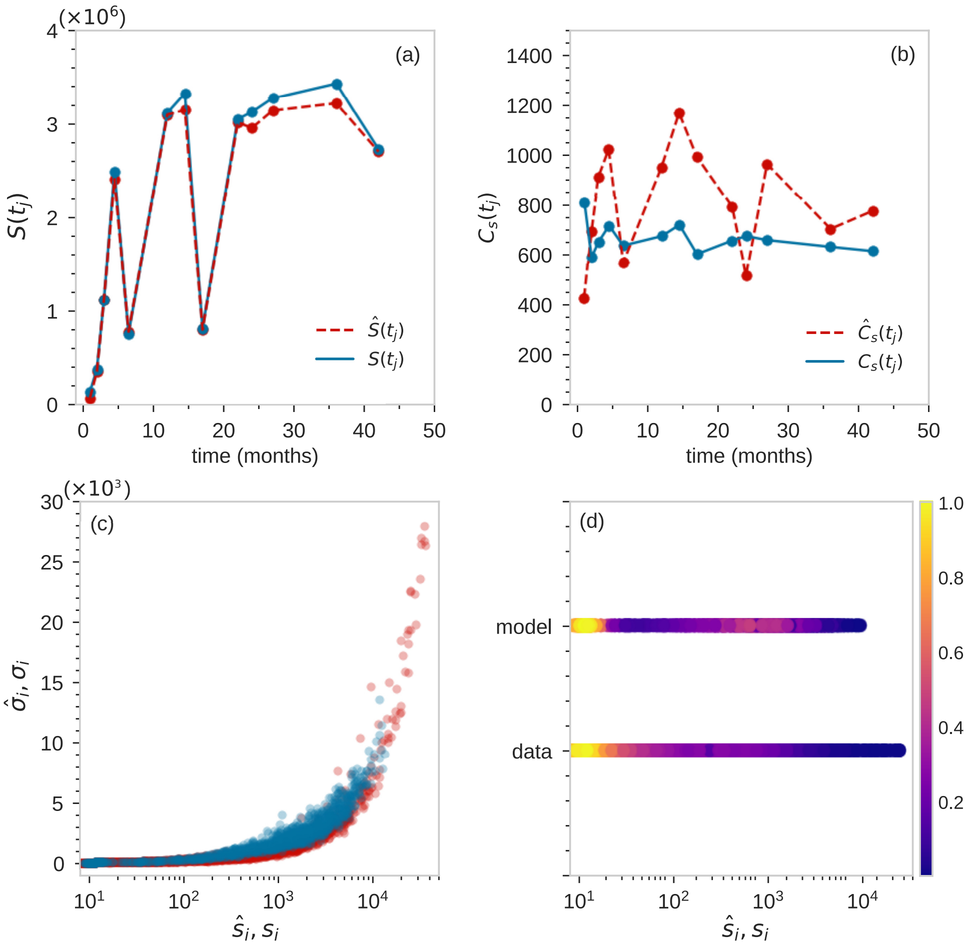

Figure 8. Fitting of tagged granulocyte populations for animal ZG66. Populations are sampled at times $t_j = [1, 2, 3, 4.5, 6.5, 12, 14.5, 17, 22, 24, 27, 36, 42]$ months. (a) By initially best-matching $S(t_j)$, we find $\eta(t_j) = [0.061, 0.354, 1.144, 2.468, 0.790, 3.176, 3.228, 0.813, 3.092, 3.029, 3.223, 3.301, 2.771] \times 10^{-4}$. (b-d) By searching parameter space to find a good match to $\hat{C}_{\rm s}(t_j), \hat{s}_i, \hat{\sigma}_i, \hat{\rho}$ (shown with maximum value normalized to one), we find $\mu_{\rm h} = 0.01, r_{\rm h}(0) = 1, C_{\rm h}(0) = 1200, C_{\rm n}(\ell = 2) = 600, K = 1.2 \times 10^5, \alpha = 0.001, r_{\rm n} = 1.5, L = 22, \mu_{\rm n} = 0, \mu_{\rm m} = 0.185$ and $\omega = 0.15$, where rates are given in units of per day.

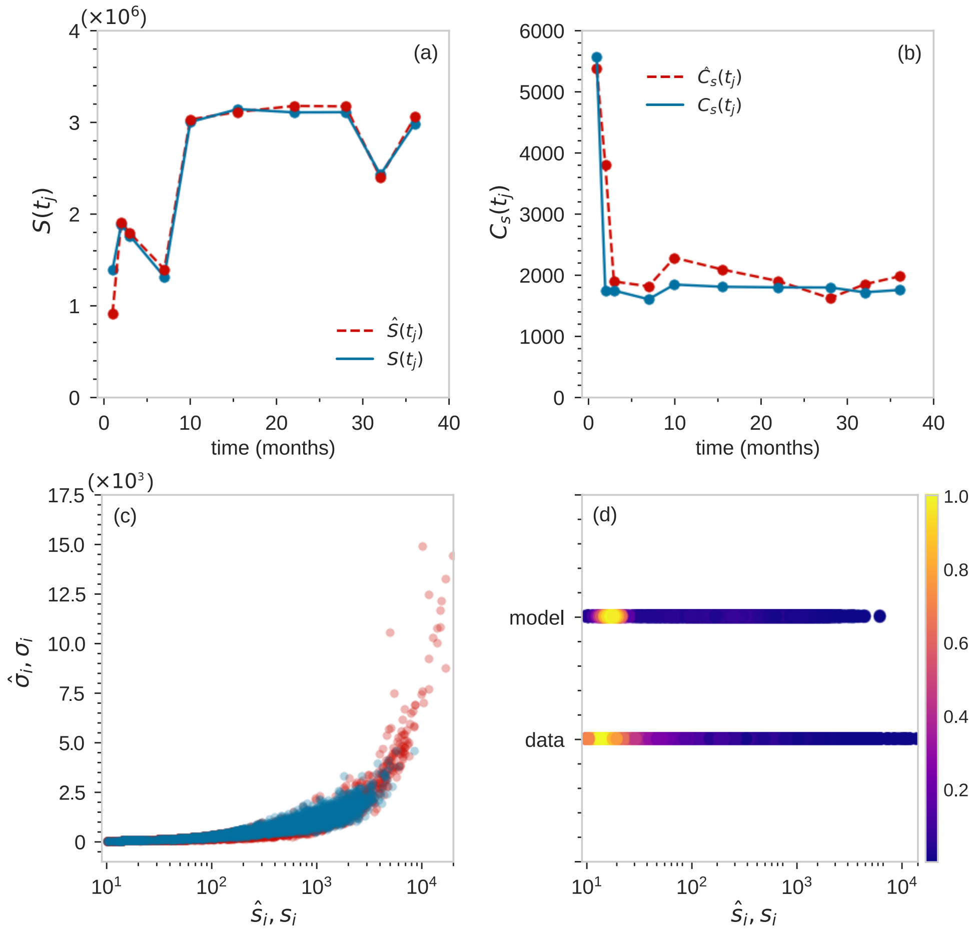

Figure 9. Fitting of tagged granulocyte populations for animal ZH19. For this animal, populations are sampled at times $t_j = [1, 2, 3, 7, 10, 15.5, 22, 28, 32, 36]$ months. (a) By initially best-matching $S(t_j)$, we find $\eta(t_j) = [0.389, 0.816, 0.764, 0.596, 1.294, 1.329, 1.357, 1.355, 1.024, 1.307] \times 10^{-4}$. (b-d) By searching parameter space to find a good match to $\hat{C}_{\rm s}(t_j), \hat{s}_i, \hat{\sigma}_i, \hat{\rho}$ (shown with maximum value normalized to one), we find $\mu_{\rm h} = 0.003, r_{\rm h}(0) = 0.02, C_{\rm h}(0) = 4000, C_{\rm n}(\ell = 4) = 4500, K = 4 \times 10^5, \alpha = 0.0011, r_{\rm n} = 1.6, L = 22, \mu_{\rm n} = 0, \mu_{\rm m} = 0.185$ and $\omega = 0.16$, where rates are given in units of per day.

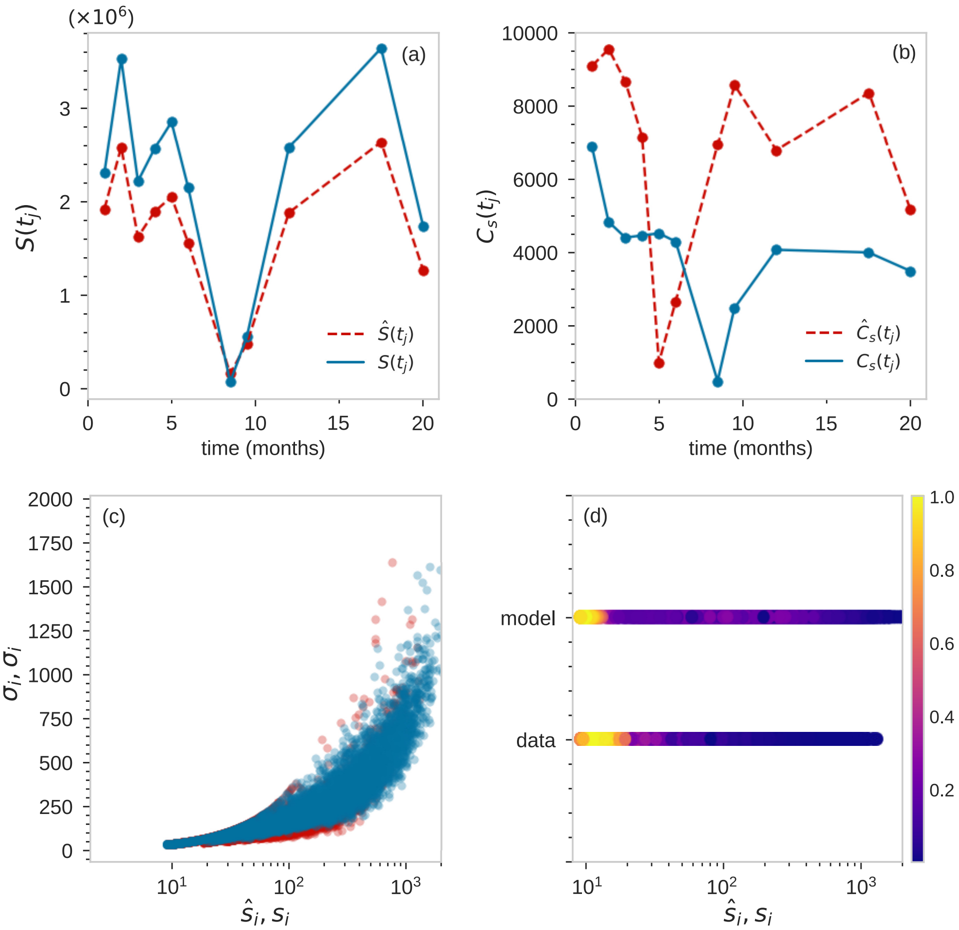

Figure 10. Fitting of tagged granulocyte populations for animal ZJ31. For this animal, populations are sampled at times $t_j = [1, 2, 4, 5, 6, 8.5, 9.5, 12, 17.5, 20]$ months. (a) By initially best-matching $S(t_j)$, we find $\eta(t_j) = [2.318, 3.118, 1.957, 2.287, 2.476, 1.879, 0.188, 0.582, 2.271, 3.186, 1.531] \times 10^{-5}$. (b-d) By searching parameter space to find a good match to $\hat{C}_{\rm s}(t_j), \hat{s}_i, \hat{\sigma}_i, \hat{\rho}$ (shown with maximum value normalized to one), we find $\mu_{\rm h} = 0.003, r_{\rm h}(0) = 0.03, C_{\rm h}(0) = 10000, C_{\rm n}(\ell = 4) = 4000, K = 10^6, \alpha = 0.0017, r_{\rm n} = 1.6, L = 22, \mu_{\rm n} = 0, \mu_{\rm m} = 0.185$ and $\omega = 0.16$, where rates are given in units of per day.
